# Detection of Circulating Tumour Cells in Urothelial Cancers and Clinical Correlations: Comparison of Two Methods

**DOI:** 10.1155/2017/3414910

**Published:** 2017-02-22

**Authors:** Emanuela Fina, Andrea Necchi, Stefano Bottelli, Carolina Reduzzi, Sara Pizzamiglio, Chiara Iacona, Maria Grazia Daidone, Paolo Verderio, Vera Cappelletti

**Affiliations:** ^1^Department of Experimental Oncology and Molecular Medicine, Fondazione IRCCS Istituto Nazionale dei Tumori, Milano, Italy; ^2^Department of Medical Oncology, Fondazione IRCCS Istituto Nazionale dei Tumori, Milano, Italy; ^3^Unit of Medical Statistics, Biometry and Bioinformatics, Fondazione IRCCS Istituto Nazionale dei Tumori, Milano, Italy

## Abstract

Circulating tumour cells (CTC) are identified exploiting their protein/gene expression patterns or distinct size compared to blood cells. Data on CTC in bladder cancer (BC) are still scarce. We comparatively analyzed CTC enrichment by AdnaTest ProstateCancerSelect (AT) and ScreenCell®Cyto (SC) kits, combined with identification by* EPCAM, MUC1, *and* ERBB2* expression and by cytological criteria, respectively, in 19 nonmetastatic (*M*_0_) and 47 metastatic (*M*_+_) BC patients, at baseline (*T*_0_) and during treatment (*T*_1_). At* T*_0_, CTC positivity rates by AT were higher in* M*_+_ compared to* M*_0_ cases (57.4% versus 25%,* p* = 0.041).* EPCAM* was detected in 75% of CTC-positive samples by AT, showing increasing expression levels from* T*_0_ to* T*_1_ (median (interquartile range, IQR): 0.18 (0.07–0.42) versus 0.84 (0.33–1.84), *p* = 0.005) in* M*_+_ cases. Overall, CTC positivity by SC was around 80% regardless of clinical setting and time point of analysis, except for a lower occurrence at* T*_1_ in* M*_0_ cases. At* T*_0_, circulating tumour microemboli were more frequently (25% versus 8%) detected and more numerous in* M*_+_ compared to* M*_0_ patients. The approach used for CTC detection impacts the outcome of CTC studies. Further investigations are required to clarify the clinical validity of AT and SC in specific BC clinical contexts.

## 1. Introduction

Circulating tumour cells (CTC) have emerged as important blood-borne biomarkers, usable for minimally invasive tissue sampling in cancer patients, in order to track the evolution of the disease in longitudinal studies and provide additional tools potentially useful in the clinical armamentarium for personalized medicine [1]. Basically, CTC can be shed from both primary and metastatic lesions [2, 3]. Baseline CTC number proved to be correlated to disease progression in many solid tumours [2], although there are indications that CTC not merely represent a surrogate of tumour burden [4, 5]. More interestingly, as CTC display phenotypic plasticity during treatment [6], assessment of their quantitative and qualitative dynamic changes and molecular features, more than CTC count itself, might represent a promising complementary application of CTC analysis [7–9]. Indeed, CTC-related biomarkers might be instrumental for the monitoring of spatiotemporal intratumour heterogeneity, allowing identification of CTC-specific gene signatures or genomic alterations emerging from resistant cellular clones, which might guide the choice of therapy [10, 11]. Despite this tempting scenario, the road to application for personalized medicine needs further efforts, and validation of CTC utility in clinical practice remains mandatory.

Full evaluation of CTC clinical validity and utility is essentially hampered by their rarity, heterogeneity, and plasticity [12, 13], three features that make CTC identification and consequently the outcome of CTC studies subject to methodological and analytical constraints and questionable reproducibility. In the perspective of clinical implementation, the development of a CTC-based test needs a careful standardization process [14, 15], but studies which compare different technical approaches are still insufficient.

At present, among the countless sophisticated technologies and bench-top devices commercially available for CTC studies, the CellSearch™ system (Janssen Diagnostic) is the only one granted FDA approval thanks to its repeatability, and it could be used as a complementary method to predict patient outcome in metastatic breast, prostate, and colorectal cancers [16, 17]. Notwithstanding the presence of CTC detected by CellSearch has been reported to be generally associated with poor prognosis in patients with solid malignancies, both at early and at advanced stages; at present no trial has yet demonstrated that modifying treatment according to CTC results provides a clinical benefit superior to the standard of care [18]. One possible reason for such a failure may be that EPCAM-based approaches (the enrichment method used in the CellSearch system) detect tumour cells which exhibit epithelial features and fail to detect subpopulations of CTC with intermediate or pure mesenchymal features [6, 19, 20]. Therefore, alternative methods allowing EPCAM-independent CTC detection, enumeration, and characterization need to be investigated.

Large interest has been recently demonstrated on the clinical significance of CTC in bladder cancer [21, 22] for which increasing incidence rates have been reported [23] and there is a pressing clinical need for new biomarkers sensitive and specific enough to parallel disease progression and treatment efficacy both in the early and in the metastatic settings. Thus, identification of patients at higher risk of relapse or progression would be beneficial for their clinical management. Data from non-CellSearch-based CTC studies are still scarce in this pathology [21, 22] and application of the liquid biopsy paradigm has been only recently explored [24].

On the basis of these considerations, we compared the performance of two distinct approaches in order to detect, count, and partially characterize CTC in bladder cancers. CTC analysis was carried out in parallel on the same samples using AdnaTest, a positive selection-dependent method, based on antibody-mediated recognition of surface markers followed by multiplex RT-PCR for epithelial or tumour-associated transcripts, and ScreenCell Cyto devices, a size-based method, which exploits physical properties for CTC enrichment and cytological analysis for detection, hence unbiased with respect to biological features of tumour cells. We also provide information on the differences in CTC detection rates and fluctuations during administration of therapy, in three different clinical settings.

To our knowledge this is the first study that compares two different technical approaches to detect CTC and to explore their applicability in different clinical contexts of bladder cancer.

## 2. Materials and Methods

### 2.1. Case Series and Donors

Patients with bladder cancer presenting to the Department of Medical Oncology at Fondazione IRCCS Istituto Nazionale dei Tumori (INT) between July 2012 and May 2014 were included. Three cohorts of patients were analyzed: 19 cases with muscle-invasive transitional cell carcinoma of the bladder (MIBC) receiving neoadjuvant therapy in a phase 2 trial of gemcitabine, cisplatin, and sorafenib (NCT01222676) and 47 patients who received systemic treatment for metastatic disease: 33 of whom received standard MVAC chemotherapy in the first-line setting [25], and 14 received second-line therapy in a phase 2 trial [26]. For patients with MIBC, blood samples were collected at baseline, at day 8 of each cycle of therapy. For patients with metastatic disease, blood samples were collected before starting a new line of treatment and at the beginning of each cycle of therapy.

Blood samples were collected from 39 healthy volunteers for CTC threshold definition and cell spiking studies. Patient and healthy donor characteristics are summarized in Table 1.

This study was carried out under an INT Review Board-approved protocol allowing the collection of biological samples from patients with genitourinary cancers and written informed consent was obtained from all patients and healthy donors.

### 2.2. Blood Sample Collection

Samples of peripheral venous whole blood were drawn from healthy volunteers or bladder cancer patients using a 21G needle and collected in 4 mL K_3_EDTA or 6 mL K_2_EDTA BD Vacutainer tubes, if intended to be processed with the AdnaTest (AdnaGen, AG, Langenhagen, Germany) or ScreenCell Cyto (ScreenCell, Sarcelles, France) kits, respectively. Samples used for CTC analysis (5 mL) were collected after withdrawal of blood volumes (roughly 50 mL) addressed to routine tests or further clinical studies, in order to minimize the risk of contamination with epithelial skin cells during puncture. Fresh samples were stored at 4°C in the dark and processed within 1 hour (for AdnaTest analysis) or 2.5 hours (for ScreenCell analysis) from withdrawal.

### 2.3. Cell Lines and Spiking Experiments

HT-1197 and RT4 bladder cancer cell lines were purchased from ATCC (Manassas, USA) and their authenticity was verified using the STR DNA profiling with the StemElite™ ID System kit (Promega, Madison, WI, USA) by INT Genomics Core Facility. HT-1197 and RT4 cells were cultured in RPMI 1640 and McCoy's 5A media (Lonza, Slough, UK), respectively, supplemented with 10% South America origin Fetal Bovine Serum (Lonza), in humidified 5% CO_2_ atmosphere.

FACS analysis for the expression of cell surface EPCAM (mouse IgG1*κ* anti- human EPCAM-PerCP-Cy7 antibody, clone 1B7, EBiosciences, San Diego, USA) and ErbB2 (mouse IgG2B anti- human ErbB2 antibody, clone 191924, R&D Systems, Minneapolis, MN, USA) was performed on fresh monodisperse RT4 cell suspensions versus isotype control samples.

For spiking experiments, highly diluted cell suspensions were prepared in culture dishes and single viable cells (detectable by Trypan blue exclusion assay) were micropipetted under an inverted optical microscope directly into conical tubes containing 5 mL of whole blood from healthy volunteer. Spiked-in samples were stored at 4°C in the dark for no more than 1 hour before processing.

### 2.4. CTC Detection by Epithelial and Tumour-Associated Antigens and Multiplex-PCR

CTC enrichment by positive selection-based method was performed using the AdnaTest ProstateCancerSelect kit. Briefly, 5 mL of whole blood per patient was incubated with 100 *μ*L of magnetic beads, coated with antibodies against the epithelial and tumour-associated antigens EPCAM and ErbB2, on a tube rotator for 25 minutes at room temperature (RT). Cell-beads complexes were captured using the AdnaMag-L magnetic particle concentrator and washed in DPBS. Cell lysates were stored at −20°C and downstream molecular analyses were performed within 2 weeks.

The expression of* EPCAM*,* MUC1*,* ERBB2*,* CEA,* and* EGFR *epithelial and tumour-specific markers was assessed by semiquantitative multiplex-PCR, following the manufacturer's instructions provided in the AdnaTest BreastCancerDetect and ColonCancerDetect kits. Briefly, mRNA was isolated using Dynabeads® Oligo(dT)_25_ and retrotranscribed in a final volume of 40 *μ*L, and two distinct multiplex-PCR were performed using BreastCancer (*EPCAM*,* MUC1*,* ERBB2 *and* ACTB*) and ColonCancer (*EPCAM*,* CEA*,* EGFR, *and* ACTB*) PrimerMixes, following each specific protocol and thermal profile. PCR products were run and solved on the Agilent 2100 Bioanalyzer (Agilent Technologies, Santa Clara, CA, USA) using the DNA 1000 kit (Agilent Technologies). The detection height threshold was set to “0” and markers' concentration expressed as “ng/*μ*L” was considered for data analysis. For quality control assessment,* ACTB* concentration ≥ 3.0 ng/*μ*L was established as necessary criterion to consider as an evaluable CTC sample, on the basis of results obtained from healthy donors with both detection kits.

### 2.5. CTC Detection by a Size-Based Approach

CTC isolation by size-based method was performed using the ScreenCell Cyto kit according to manufacturer's instructions. Briefly, two 2.5 mL aliquots of whole blood per patient were separately mixed with 4 mL of a proprietary red blood cell lysis and fixation buffer and incubated for 8 minutes at RT. Samples were filtered on two distinct isolation devices and CTC were isolated exploiting circular pores randomly distributed throughout the isolation supports (IS),* that is*, a microporous membrane. IS were rinsed in DPBS, air-dried, and immediately stained with Hematoxylin Solution S (Merck, Darmstadt, Germany) for 1 minute and then with Shandon Eosin Y Aqueous Solution (Thermo Fisher Scientific Inc., Waltham, MA, USA) for 30 seconds, at RT. Samples were stored at −20°C until cytological evaluation by a certified pathologist.

Single CTC or circulating tumour microembolus (CTM) were identified on the basis of previously reported cytopathological criteria for malignancy [27]. Major criteria were nuclear size ≥ 20 *μ*m and nuclear-to-cytoplasmic ratio ≥ 0.75, whereas irregular nuclear contours and nuclear hyperchromatism where considered among minor criteria. CTM were defined as clusters of at least two CTC (including those surrounded by platelets and fibrin), showing criteria of malignancy like those described for single CTC. Samples showing poor quality of cytology, estimated on the basis of poor preservation of the leukocytes, were excluded from the analysis. Samples were rated as CTC or CTM positive if at least 1 CTC or CTM was detected in at least one of the two IS. Results were expressed as total CTC and CTM numbers when both IS (corresponding to 5 mL of blood) were evaluable, according to the quality of cytology.

### 2.6. Statistical Analysis

The technical reliability of AdnaTest approach was assessed in ex vivo spiking experiments with bladder cancer cell lines. Different quantities (0-10-20-40) of RT4 cells (99.9% EPCAM^+^ErbB2^+^ by FACS analysis) were spiked in 5 mL of whole blood samples and processed using AdnaTest. In parallel, the same amounts of cells were spiked in lysis buffer as a reference for capture yields evaluation and directly processed for molecular analysis. The pattern of the relationship between the concentration of* EPCAM(B)* (resulted less variable compared to* EPCAM(C) *(data not shown))*, ERBB2,* and* EGFR* markers measured from the two different extraction matrixes blood and lysis buffer was then evaluated by resorting to linear regression approaches and the adequacies of the models were assessed by R square index [28]. The identification the optimal cutoff for each marker was based on receiver operating characteristic (ROC) methodology by maximizing the corresponding Youden index [29]. The outcome provided by the two CTC detection approaches was dichotomized, and the degree of concordance was assessed by calculating the prevalence-adjusted and bias-adjusted kappa (PABAK) statistics and its 95% confidence interval [30] and interpreted on the basis of the Landis and Koch classification criteria [31]. The relationship between each considered continuous variable (CTC and CTM count, markers concentration) and the clinical settings (*M*_0_, *M*_1_, and *M*_2_) were investigated by mean of Kruskal-Wallis test [32]. Fisher exact test was used to assess the performance of each considered CTC detection approach according to the clinical setting.

All statistical analyses were carried out with the SAS (Version 9.2.; SAS Institute, Inc., Cary, NC) and R software by adopting a significance level of *α* = 0.05.

## 3. Results

### 3.1. Assessment of the Technical Reliability of the Two CTC Detection Approaches

As concerns the technical reliability of AdnaTest approach, we observed a linear relationship between the concentration values of each marker obtained from spiked blood samples undergoing capture and those of an equivalent number of pure RT4 cells, with *R*^2^ indexes equal to 0.81, 0.86, and 0.96 for* EPCAM*,* ERBB2,* and* EGFR*, respectively (*MUC1* and* CEA* were not detectable in RT4 cells). These results suggest that AdnaTest's efficacy in capturing tumour cells is acceptable. On the other hand, the median recovery by ScreenCell approach for 0-1-5-10-25 tumour cells, spiked in 2.5 mL of blood, was 90% (range 0–100%). Single cell recovery was successful in 1 out of 2 independent experiments. No tumour cells were detected in healthy donors (4 individuals).

### 3.2. Cutoff and Criteria for CTC Positivity Assignment by AdnaTest

Considering that the AdnaTest approach allows indirect assessment of the presence of CTC and that baseline signals can be obtained from noncancerous cells attached to magnetic beads in nonspecific manner, positivity cutoffs were calculated by ROC curve analysis on data obtained from 39 healthy subjects and 29 patients before starting first-line chemotherapy for metastatic disease (Table 1). Compared to healthy donors,* EPCAM*,* MUC1,* and* ERBB2* expression levels were higher in patients, and the areas under the ROC curves were 0.596 for* EPCAM*, 0.608 for* MUC1,* and 0.552 for* ERBB2*. Optimal cutoff maximizing sensitivity and specificity (Youden index) were estimated at ≥0.37 ng/*μ*L (round off to 0.40) for* EPCAM*, ≥0.10 ng/*μ*L for* MUC1, *and ≥0.18 ng/*μ*L (round off to 0.20) for* ERBB2*.* CEA* and* EGFR* levels were all 0 in the healthy donor group; for this reason positivity cutoff equivalent to technical detection threshold (0.10 ng/*μ*L) was assigned to both markers.

As the number of positive samples in controls (healthy donors, 8/39) and cases (patients, 16/29) was similar using either combination or at least one of the three markers among* EPCAM*,* MUC1,* and* ERBB2* (positivity did not change when combining aforementioned markers with* CEA *and* EGFR*), samples were considered as CTC positive when at least one among* EPCAM*,* MUC1,* and* ERBB2 *markers was higher than the defined cutoff value. Samples with all markers' concentration under the cutoff values were defined as CTC negative.

### 3.3. Analysis of CTC Positivity by AdnaTest within the Clinical Settings

At baseline, CTC positivity was lower in *M*_0_ (25%) compared to *M*_+_ cases (i.e., patients under first-line therapy, hereafter referred to as “*M*_1_,” and second-line therapy, hereafter referred to as “*M*_2_,” 57.4%, *p* = 0.041), with a similar trend also at *T*_1_ (60% versus 86.5%,* p* = 0.058). In *M*_0_ cases, positivity by AdnaTest rose from 25 to 60% after the first cycle of therapy then reaching a steady state after the second cycle, whereas in *M*_+_ cases the increasing trend of CTC positivity was less apparent and mainly evident from *T*_0_ to *T*_1_ samples (Table 2).

**Figure 1:**
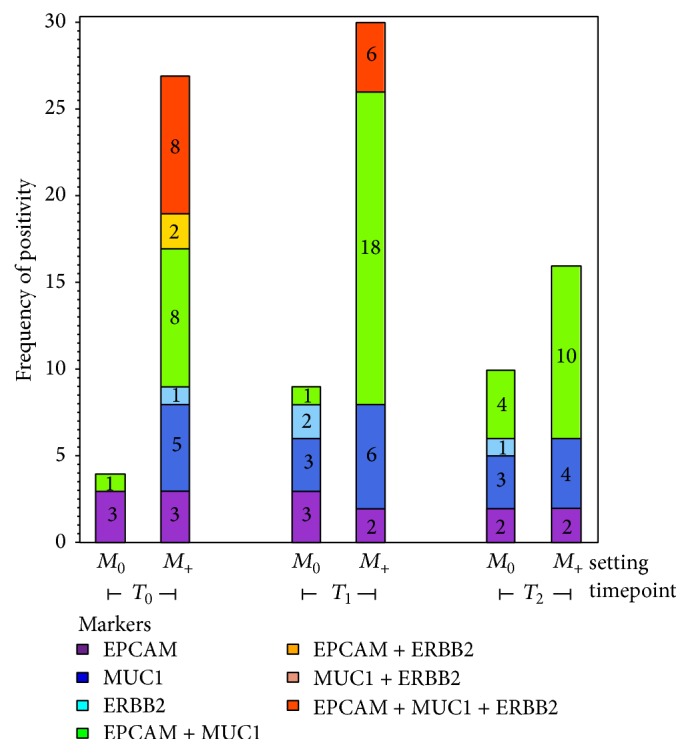
Contribution of AdnaTest markers to circulating tumour cell (CTC) positivity. The contribution of* EPCAM*,* MUC1*,* ERBB2* and of all their combinations to CTC positivity is reported for *M*_0_ and *M*_+_ cases at each time point analyzed (baseline, *T*_0_; after first, *T*_1_; and second, *T*_2_, cycle of therapy). Numbers within the bars refer to the number of CTC-positive samples expressing specific markers or their combinations as indicated by the colour code.

Analysis of CTC molecular features by AdnaTest revealed a different contribution of* EPCAM*,* MUC1,* and* ERBB2* markers to CTC positivity, according to the specific clinical settings and time points (Figure 1).* EGFR* and* CEA* were not considered in this analysis as they were seldom expressed. In *M*_0_ cases* EPCAM* was the most represented marker in CTC+ samples (4/4 cases), and was found to be coexpressed with* MUC1* in 1 out of 4 samples. In *M*_+_ cases the main contribution to CTC positivity derived from the presence of at least one marker between* EPCAM *and* MUC1* either alone or in combination with other markers, both at baseline and during therapy.* ERBB2* expression was observed in *M*_+_ patients both at *T*_0_ and *T*_1_, although not as single marker, but only during therapy in *M*_0_ patients. Interestingly,* EPCAM* and* MUC1* were found to be more frequently coexpressed in CTC+ samples collected during therapy in *M*_+_ compared to *M*_0_ cases. Coexpression of the three markers was never detected in *M*_0_ cases and was instead observed in *M*_+_ cases both at baseline and at *T*_2_.

**Figure 2:**
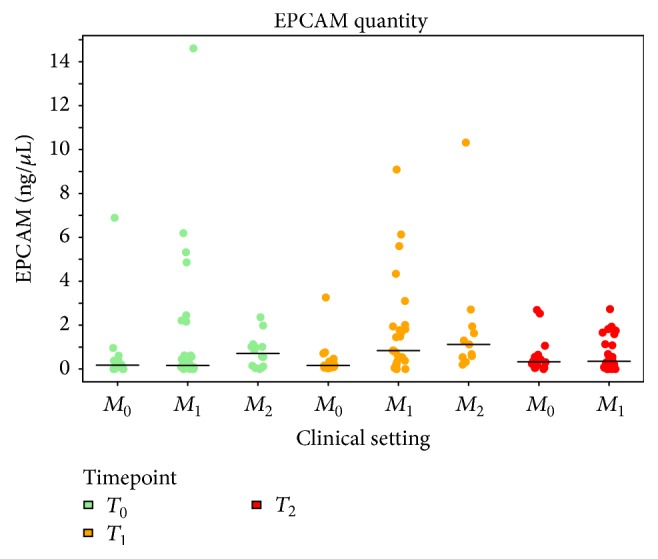
EPCAM expression in the different clinical settings. EPCAM levels (ng/*μ*L) by RT-PCR in samples processed by AdnaTest approach are reported for each clinical setting (patients with muscle-invasive nonmetastatic bladder cancer, *M*_0_; patients with *M*_+_ bladder cancer under first-line, *M*_1_, or after second-line, *M*_2_, therapy) and for each time point analyzed: baseline (*T*_0_, green dots), after the first (*T*_1_, yellow dots), and after the second (*T*_2_, red dots) cycle of therapy.

Concentration of* EPCAM*, the most frequently detected marker in AdnaTest positive samples (overall 75% at baseline), was considered as a surrogate marker of CTC burden and associated with tumour stage at different time points (Figure 2).* EPCAM* levels increased 4-fold from baseline to *T*_1_ in samples from *M*_+_ patients (median for *T*_0_, 0.18; interquantile range [IQR], 0.07–0.42 versus median for *T*_1_, 0.84; IQR, 0.33–1.84;* p* = 0.005), while they did not change following neoadjuvant treatment in *M*_0_ patients (median for *T*_0_, 0.18; IQR, 0.09–1.01; median for *T*_1_, 0.16; IQR, 0.06–0.47). At baseline,* EPCAM* levels were comparable in samples from *M*_0_ and *M*_+_ patients (median for *M*_0_, 0.18; IQR, 0.07–0.42; median for *M*_+_, 0.18; IQR, 0.09–1.01).* MUC1* concentrations were similar at *T*_0_ and *T*_1_ in *M*_0_ cases (0.04 (0.00–0.07) and 0.03 (0.00–0.10)), whereas a 5-fold increase from *T*_0_ to *T*_1_ was observed in *M*_+_ cases (0.08 (0.05–0.28) versus 0.42 (0.19–0.73)).* ERBB2* levels were similar at any time point and in each setting, with overall median (IQR) concentrations 0.04 (0.01–0.12) for *T*_0_ and 0.06 (0.00–0.15) for *T*_1_.

### 3.4. Analysis of CTC Positivity by ScreenCell within the Clinical Settings

**Figure 3:**
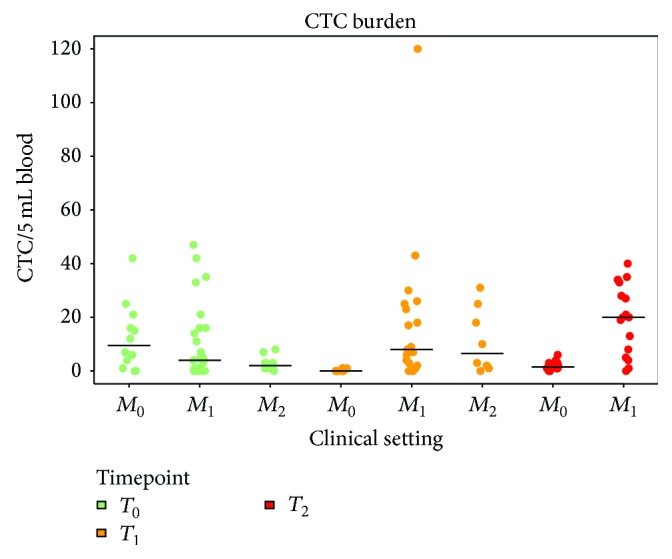
Circulating tumour cell (CTC) distribution in the different clinical settings. The number of CTC per 5 mL of blood, detected by ScreenCell approach, is reported for each clinical setting (patients with muscle-invasive nonmetastatic bladder cancer, *M*_0_; patients with *M*_+_ bladder cancer, under first-line, *M*_1_, or second-line, *M*_2_, therapy) at each time point analyzed: baseline (*T*_0_, green dots), after the first (*T*_1_, yellow dots), and after the second (*T*_2_, red dots) cycle of therapy.

Using the size-based ScreenCell approach and a positivity cutoff of at least 1 CTC or CTM, 80% of cases were defined as CTC-positive, regardless of the clinical setting and the time point of analysis, except for a lower occurrence at *T*_1_ in *M*_0_ cases (Table 3). Notwithstanding such findings, enumeration of CTC revealed no statistically significant (*p* = 0.191) lower CTC count at baseline in *M*_+_ (median 3; IQR, 1–9.5) compared to *M*_0_ (median 9.5; IQR, 2.5–18.5) patients. Conversely, after the first cycle of therapy, CTC numbers appeared significantly (*p* = 0.0004) increased in *M*_+_ cases (median, 8; IQR, 2–23) compared to baseline data, and a similar trend was also observed after the second cycle of therapy (median 20; IQR, 6.5–30.5*; p* = 0.0003), while a trend toward a decrease, although not significant, was observed in *M*_0_ cases (median 0; IQR, 0-1 at *T*_1_ and median 1.5; IQR, 1–3 at *T*_2_) (Figure 3).

CTM counts were also considered and, despite their rarity compared to the population of single CTC (overall at *T*_0_ they represent the 25%,* that is*, 10/40 CTC-positive samples), at baseline they were more frequently detected in *M*_+_ compared to *M*_0_ cases (9/36, 25% versus 1/12, 8%) and their median and IQR numbers in CTM-positive *M*_+_ cases were 4 (2–19) compared to 1 CTM found in the only CTM-positive *M*_0_ case.

### 3.5. Concordance between AdnaTest and ScreenCell

The concordance between AdnaTest (AT) and ScreenCell (SC) in detecting CTC was assessed on matched samples belonging to the three different clinical settings collected at *T*_0_ and *T*_1_. As reported in Table 4, the proportion of concordant samples (AT/SC both positive or negative) was around 30% in patients with early stage disease (*M*_0_ setting), both at baseline and during therapy, whereas higher interassay concordances were observed in patients with metastatic disease, mainly following treatment. The overall, concordance between the two technical approaches was however poor, both at baseline (prevalence-adjusted bias-adjusted kappa, PABAK = 0.020, 95% CI 0.019–0.059) and during therapy (PABAK = 0.282, 95% CI 0.235–0.329).

The direction of the discordance was investigated considering CTC positivity rates in samples processed by both methods (Table 4). Overall, at baseline, more samples were called positive by SC compared to AT (84.3 versus 54.9%), with differences between *M*_0_ and *M*_+_ (overall *M*_1_ and *M*_2_) cases (84.6 versus 30.8%, and 84.2 versus 63.2%, resp.). Discrepancies on CTC positivity ratings by AT and SC appeared to be also influenced by therapy administration, since it decreased after the different treatment cycles with the increase of samples called CTC-positive by AT.

## 4. Discussion

The results of this study suggest that in urothelial tumours, similarly as in other tumours [33–37], the outcome of CTC studies is markedly influenced by the specific technical approach chosen for CTC assessment. In fact, the ScreenCell approach called CTC positive more than 80% of the cases, independently from the stage of disease, whereas AdnaTest failed to detect CTC in about 40% of metastatic patients, despite systemic dissemination being expected to take place in the vast majority of cases. Moreover, in a matched comparison, its detection rate in patients with early stage disease was about 3 times lower compared to ScreenCell. The ability of AdnaTest to discriminate between healthy subjects and patients was modest (AUC around 0.60) when using EPCAM and ERBB2 as surface antigens for CTC enrichment and classical epithelial- or tumour-associated markers (*EPCAM, MUC1, *and* ERBB2*) for detection, thus providing an explanation for different CTC detection rates and scarce concordance when comparing the two methods. A possible further application could be to consider the two investigated methods in a complementary scenario by developing, for example, an algorithm based on their sequential usage in order to better characterize CTC positivity status for each patient. For AdnaTest the global contribution to CTC positivity at baseline was driven by* EPCAM *(19.4%),* MUC1 *(16.1%), and their combination (29.0%), whereas the contribution of* ERBB2* was not relevant considered either alone (3.2%) or in combination with* EPCAM* (6.5%).* EPCAM *and* MUC1 *were the most represented markers among CTC-positive samples especially in patients with metastatic disease, where positivity for* CEA* and* EGFR* was also observed though at low frequency. Conversely in *M*_0_ cases only* EPCAM *contribution was determinant for defining CTC positivity. CTC load directly assessed either by CTC count or by considering* EPCAM *concentrations as surrogate marker did not appear to reflect tumour burden.

CTM were found to account for a small portion of the global CTC population, in agreement with recent observations in experimental models [38] and clinical samples [39]. Moreover, they were more frequently detected in *M*_+_ compared to *M*_0_ cases, suggesting higher metastatic potential and possible prognostic role also in metastatic urothelial cancers, as already reported for other tumour types [36, 39–42]. Similar results have been reported by Anantharaman et al. [43] who were able to identify by cytokeratin immunostaining CTC clusters in 6/21 (28.6%) metastatic cases and in none of four nonmetastatic bladder tumours.

As suggested by fluctuations in CTC positivity rates measured with both methods, it is possible that treatment-induced modifications in CTC phenotypes might have impacted the test performance, and that drugs with different mechanisms of action might affect CTC dynamics, mainly when detection refers on the expression of functional markers. In *M*_0_ cases, analysis by AdnaTest revealed an opposite trend compared to ScreenCell, as CTC positivity increased after chemotherapy for the former, whereas a stepwise decrease was observed by CTC count at each cycle of therapy. The AdnaTest result could be interpreted in different ways: it may represent an increased expression by single CTC without a change in the global CTC number, or on the opposite as an increased fraction of CTC expressing epithelial markers. We also cannot exclude the notion that chemotherapy might have triggered the release of bone marrow derived edonthelial precursors in the peripheral blood,* that is*, of cells that to a certain degree express epithelial markers but lose the expression of hematopoietic antigens [44]. However, such a hypothesis, although fascinating, does not seem to be supported by the observation of an increased concordance between AT and SC after chemotherapy as reported in Table 4, since bone marrow-derived precursor cells would not be identified as CTC by the morphological criteria adopted in SC. Moreover, neoadjuvant therapy before cystectomy was proved to provide clinical benefit in patients with MIBC [45], and ScreenCell-defined CTC fluctuations seem indeed to support the good treatment response. An alternative possible interpretation could be a low expression or absence of epithelial markers in CTC in nonmetastatic disease at baseline, leading to false negatives.

In patients with metastatic disease CTC positivity by AdnaTest increased after the first cycle of therapy and reached percentage value similar to baseline data after the second cycle, while CTC positivity given by their direct count was high and similar at any time point. Also in this case CTC trend was quite different and groups of distinct patients showing persistent positive or negative CTC status by AdnaTest were identified, but correlations with clinical outcome are required to understand their significance.

CTC data interpretation appears to be more straightforward in metastatic patients receiving second-line therapy, where overall CTC levels, assessed either by AdnaTest or by ScreenCell, increased after therapy administration, thus mirroring the lack of response observed in this cohort [26].

At present, excluding studies with CellSearch system, detection of CTC in patients with bladder cancer is based on the expression of different epithelial markers at mRNA level in mononuclear cells isolated from 5 to 12 mL of peripheral venous blood, as reported for EGFR, CK-19 and CK-20 [46–51], and tumour-associated markers as MUC-7, Tenascin C, and Survivin [51, 52], with CTC positivity rates ranging from 10 to 80%, according to the type of marker and the stage of disease.

Recently, the use of improved AdnaTest immune-magnetic systems also allowed the identification of CTC with stem-like features by PCR-based methods, showing that* ALDH1 *mainly contributed to CTC positivity compared to* EPCAM*,* MUC1,* or* ERBB2 *in bladder cancer patients [53].

From a biological point of view, in metastatic cases CTC are expected to revert to the epithelial phenotype through mesenchymal-to-epithelial transition process in order to colonize distant sites, whereas they should tend to maintain mesenchymal rather than epithelial features during dissemination at early stages of disease. This biological consideration might account for the discrepancy in CTC detection rates in *M*_0_ cases observed between our AdnaTest-based approach and ScreenCell, since the latter is unbiased regarding the biological phenotype of the cell. Nevertheless, data on the role of epithelial-to-mesenchymal transition in dissemination and formation of secondary tumours are still controversial [54].

The advent of semiautomatic technologies such as the CellSearch system has allowed evaluation of the prognostic role of CTC status in patients with nonmetastatic bladder cancer [55–57], generally showing reduced disease-free survival when CTC are present, but data on the clinical role of CTC in metastatic disease are still lacking. Recent advancements in engineering and molecular biology techniques positively influenced CTC studies, which frequently entered clinical trials in the latest years. However, optimal conditions and approaches for CTC enrichment, detection, and analysis still remain to be established. Nevertheless, standardization and analytical validation steps as well as consensus-based metrics for determining limits of CTC measurement have not yet been defined at present. The number of studies addressing these issues is still limited.

## 5. Conclusions

In our study we originally developed an analytical method for CTC detection in bladder cancer, through an approach based on multiplex RT-PCR of a panel of tumour molecular markers and immunomagnetic enrichment. In addition, our study is the first one that compares the performance of two distinct CTC tests in different clinical settings of urothelial cancers, using parallel blood samples collected both at baseline and at different cycles of systemic therapy. Possible limitations of the study are represented by quantity and quality of markers chosen to capture and characterize CTC, but also by the lack of biological information on CTC isolated by filters. Both biological and technical variability could have influenced the test performance, since CTC might change their phenotype during hematogenous dissemination, losing some epithelial markers, and the panel of surface antigens beyond EPCAM, as ERBB2, MUC1, EGFR, and HGFR, currently exploited to identify CTC by immunological methods, might still be incomplete. On the other hand, morphological criteria are thought to be not sufficient to identify CTC [58] and in some cases the use of specific markers might help in distinguishing them from tumour-associated hematopoietic cells, as cancer-associated macrophages-like cells [59] or tumour-associated neutrophils [60]. The approaches proposed in this study require to be further investigated to analyze CTC in urothelial cancers. Whether the proposed methods will be applicable to monitor therapy response still remains to be determined in future studies.

## Figures and Tables

**Table 1:** Clinical and demographic characteristics for the four study groups (MIBC, metastatic patients submitted to first-line and second-line therapy and healthy donors).

Variable	Group			
	Newly diagnosed, receiving neoadjuvant chemotherapy (19 cases)	Metastatic, receiving 1st-line chemotherapy (33 cases)	Metastatic, receiving 2nd-line therapy (14 cases)	Healthy donors (39 cases)
*Sex*				
Female	4 (21.1%)	10 (30.3%)	1 (7.1%)	17 (43.6%)
Male	15 (78.9%)	23 (69.7%)	13 (92.9%)	22 (56.4%)
*Age*				
<65	16 (84.2%)	14 (42.4%)	6 (42.9%)	39 (100%)
≥65	3 (15.8%)	19 (57.6%)	8 (57.1%)	0
*Primary tumour site*				
Bladder	19 (100%)	28 (84.8%)	11 (78.6%)	—
Upper tract	0	5 (15.2%)	3 (21.4%)	—
*Metastatic sites*				
Lymph-nodes		22 (66.7%)	12 (85.7%)	—
Liver-lung-bone		13 (39.4%)	9 (64.3%)	—
Other^*∗*^		5 (15.2%)	1 (7.1%)	—
*Histology*				
Pure TCC	2 (10.5%)	24 (72.7%)	13 (92.9%)	—
Other^†^	17 (89.5%)	7 (21.2%)	1 (7.1%)	—
*Smoking habit* ^§^				
Never smoker	8 (42.1%)	9 (27.3%)	2 (14.3%)	9 (23.1%)
Former smoker	7 (36.8%)	12 (36.4%)	3 (21.4%)	5 (12.8%)
Current smoker	4 (21.1%)	10 (30.3%)	3 (21.4%)	25 (64.1%)

MIBC: muscle-invasive bladder cancer; TCC: transitional cell carcinoma.

^*∗*^Peritoneum, bladder.

^†^TCC + sarcomatoid, small cell differentiation, squamous cell.

^§^Missing data in 2 out of 33 cases.

**Table 2:** CTC positivity (number of cases and percentage) by AdnaTest stratified according to clinical settings and time of analysis.

Clinical setting	CTC+ (%) at baseline (*T*_0_)	CTC+ (%) after the 1st cycle of therapy (*T*_1_)	CTC+ (%) after the 2nd cycle of therapy (*T*_2_)
*M*_*0*_	4/16 (25.0%)	9/15 (60.0%)	10/16 (62.5%)
*M*_*+*_	27/47 (57.4%)	32/37 (86.5%)	16/25 (64.0%)
*M*_1_	*18/33 (54.6%)*	*21/26 (80.8%)*	*16/25 (64.0%)*
*M*_2_	*9/14 (64.3%)*	*11/11 (100%)*	—
*Total*	31/63 (49.2%)	41/52 (78.8%)	26/41 (63.4%)

**Table 3:** CTC positivity (number of cases and percentage) by ScreenCell stratified according to clinical settings and timepoints.

Clinical setting	CTC+ (%) at baseline (*T*_0_)	CTC+ (%) after the 1st cycle of therapy (*T*_1_)	CTC+ (%) after the 2nd cycle of therapy (*T*_2_)
*M*_*0*_	11/13 (84.6%)	4/10 (40.0%)	12/14 (85.7%)
*M*_*+*_	32/38 (84.2%)	27/30 (90.0%)	16/17 (94.1%)
*M*_1_	*22/27 (81.5%)*	*20/22 (90.9%)*	*16/17 (94.1%)*
*M*_2_	*10/11 (90.9%)*	*7/8 (87.5%)*	—
*Total*	43/51 (84.3%)	31/40 (77.5%)	28/31 (90.3%)

**Table 4:** Concordance between CTC positivity evaluated by AdnaTest (AT) and Screen Cell (SC) on the same samples and as a function of the line of treatment.

Clinical setting	Before therapy				After therapy			
	*N*	CTC+ by AT (%)	CTC+ by SC (%)	Concordant samples (%)	*N*	CTC+ by AT (%)	CTC+ by SC (%)	Concordant samples (%)
*M*_0_	13	4 (30.8)	11 (84.6)	4 (30.8%)	10	5 (50.0)	4 (40.0)	3 (30.0%)
*M*_1_	27	16 (59.3)	22 (81.5)	15 (55.6%)	21	17 (81.0)	19 (90.5)	15 (71.4%)
*M*_2_	11	8 (72.7)	10 (90.9)	7 (63.6%)	8	8 (100.0)	7 (87.5)	7 (87.5%)
*Total*	51	28 (54.9)	43 (84.3)	26 (50.9%)	39	30 (76.9)	30 (76.9)	25 (64.1%)
